# Correction: Guidelines for Accurate and Transparent Health Estimates Reporting: the GATHER statement

**DOI:** 10.1371/journal.pmed.1002116

**Published:** 2016-08-09

**Authors:** Gretchen A. Stevens, Leontine Alkema, Robert E. Black, J. Ties Boerma, Gary S. Collins, Majid Ezzati, John T. Grove, Daniel R. Hogan, Margaret C. Hogan, Richard Horton, Joy E. Lawn, Ana Marušić, Colin D. Mathers, Christopher J. L. Murray, Igor Rudan, Joshua A. Salomon, Paul J. Simpson, Theo Vos, Vivian Welch, The GATHER Working Group

The affiliation for the 18^th^ author is incorrect. Theo Vos is not affiliated with #12 but with #11 Institute for Health Metrics and Evaluation, University of Washington, Seattle, Washington, United States of America.
